# Photobiomodulation in animal models of ophthalmic disease: protocol for systematic review and meta-analysis of morphological, visual function, and inflammatory outcomes

**DOI:** 10.3389/fmed.2026.1904150

**Published:** 2026-08-12

**Authors:** Jin Mi Chun, Seunghwan Choy, Ji-Woo Seok, Jung-Dae Kim, Se-Ra Park

**Affiliations:** 1 Digital Health Research Division, Korea Institute of Oriental Medicine, Daejeon, Republic of Korea; 2 Korean Medicine Convergence Research Division, Korea Institute of Oriental Medicine, Daejeon, Republic of Korea

**Keywords:** age-related macular degeneration, corneal injury, diabetic retinopathy, glaucoma, meta-analysis, ophthalmic diseases, photobiomodulation, preclinical animal models

## Abstract

**Background:**

Despite accumulating preclinical evidence supporting the therapeutic efficacy of photobiomodulation (PBM) in ophthalmic disease models, the systematic evaluation and quantitative synthesis of these findings remain underexplored. This protocol aims to comprehensively synthesize existing studies by categorizing outcome measures into morphological, visual, and inflammatory domains according to ophthalmic pathology. The primary objective of this study is to evaluate the therapeutic efficacy of PBM for ophthalmic diseases and to consolidate the current body of preclinical evidence.

**Methods:**

This study will be conducted in accordance with the Preferred Reporting Items for Systematic Reviews and Meta-Analysis Protocols (PRISMA-P) guidelines. We will include controlled preclinical studies using animal models of four predefined ophthalmic conditions: age-related macular degeneration (AMD), diabetic retinopathy (DR), glaucoma, and corneal injury. A systematic search will be conducted across relevant electronic databases without restrictions on publication date. Outcomes will be categorized into three domains, morphological, visual functional, and inflammatory, to evaluate the efficacy of PBM in ophthalmic diseases. Two independent reviewers will perform study selection, data extraction, and risk of bias assessment using the SYRCLE tool. Where appropriate, meta-analyses will be conducted using random-effects models, with heterogeneity assessed using I^2^ statistics. Subgroup and sensitivity analyses will be performed to explore sources of variability.

**Discussion:**

This systematic review aims to integrate and assess the quality of current evidence regarding PBM’s efficacy across morphological, visual functional, and inflammatory outcomes. Findings will contribute to future trial designs and support the development of standardized protocols for PBM applications in ophthalmology.

**Systematic review registration:**

https://www.crd.york.ac.uk/PROSPERO/view/CRD420251054002, PROSPERO CRD420251054002.

## 1 Background

Ophthalmic diseases encompass a wide range of conditions affecting the structure and function of the eye, including the retina, optic nerve, and anterior segment. These conditions can cause decreased vision, visual field defects, pain, inflammation, and potentially blindness (1). The prevalence and incidence of ophthalmic diseases vary by disease type, country, and age group; however, the burden of these diseases continues to increase worldwide owing to aging and an increase in chronic diseases (2). According to the World Health Organization, more than 2.2 billion people globally are affected by some form of vision impairment or blindness. This number is expected to increase further owing to population growth, aging, lifestyle changes, and increased urbanization. The global burden of visual impairment and blindness poses significant public health challenge (3). The prevalence of age-related eye diseases, such as age-related macular degeneration (AMD), glaucoma, and diabetic retinopathy (DR), is particularly high among the elderly, and these conditions are recognized as major causes of blindness. In addition to corneal injuries, ophthalmic diseases are the leading causes of visual impairment and blindness worldwide. Among these, glaucoma and DR have been the most extensively studied because of their high prevalence, significant risk of blindness, and complex pathophysiology (3, 4).

Currently, several Food and Drug Administration-approved therapies exist for major ophthalmic diseases, including anti-vascular endothelial growth factor (VEGF) therapy for AMD and DR, topical intraocular pressure-lowering agents for glaucoma, and even gene therapy for inherited retinal dystrophies (5, 6). Despite advances in pharmacological and surgical treatments, many of these diseases remain chronic and progressive, with limited options for early intervention and neuroprotection. Moreover, current therapies are often invasive, require repeated administration, and primarily focus on slowing disease progression rather than restoring visual function (7, 8). Given these therapeutic limitations, photobiomodulation (PBM), a non-invasive technique that applies low-intensity red light to near-infrared light to stimulate cellular activity, has garnered increasing attention as a promising alternative treatment strategy (3, 8).

In addition to low-level light therapy (LLLT), PBM is primarily recognized for its ability to enhance mitochondrial function, reduce oxidative stress, and modulate inflammation-associated signaling pathways (9–11). PBM has demonstrated therapeutic efficacy in neurological and musculoskeletal disorders and its effectiveness has been increasingly validated in ophthalmic diseases (12–14). In ophthalmology, preclinical studies have demonstrated that PBM confers a range of therapeutic benefits, including attenuation of retinal degeneration, preservation of photoreceptor and ganglion cell populations, and improvement of visual behavioral outcomes in models of AMD, DR, glaucoma, and corneal injury (15–18). Given its non-invasive nature, lack of tissue damage or cumulative toxicity, and minimal risk of systemic or immunological adverse effects compared to pharmacological or surgical interventions, PBM is regarded as a particularly promising therapeutic strategy for the long-term treatment of chronic and progressive ophthalmic diseases (14, 19).

Recently, the Valeda Light Delivery System was approved by the U.S. and FDA as the first PBM device for the treatment of dry AMD (5, 20). This demonstrates the growing interest in PBM as a promising noninvasive treatment option for chronic ophthalmic diseases. In addition, PBM devices for glaucoma, DR, and corneal injury are currently under development and investigation (21).

However, fundamental preclinical data supporting the efficacy of PBM in ophthalmic diseases remain fragmented and underdeveloped, despite growing clinical interest and technological improvements. Considering this gap, it is necessary to thoroughly evaluate preclinical studies to determine whether the biological effects of PBM identified in preliminary research warrant further clinical investigation.

Preclinical animal studies play a critical role in bridging the gap between basic research and clinical application by providing a foundational platform for elucidating disease mechanisms and evaluating therapeutic efficacy (22, 23). In ophthalmology, animal models enable the controlled investigation of retinal, optic nerve, and corneal pathologies, facilitating the reproducible induction of disease-specific phenotypes, such as photoreceptor degeneration, retinal vascular abnormalities, optic nerve damage, and corneal injury. These models offer a rigorous experimental framework for quantifying the structural, functional, and molecular outcomes of therapeutic interventions such as PBM. Recent preclinical studies have reported the promising therapeutic effects of PBM in various ophthalmic disease models. For instance, in models of photo-oxidative retinal injury, complement factor H (CFH) knockout mice, and diabetic retinopathy, PBM has been shown to preserve photoreceptor cells, reduce oxidative stress, inhibit microglial activation, and enhance ATP production (14, 24).

Despite these promising findings, the interpretability and applicability of existing evidence remain limited. Existing preclinical studies applying PBM to ophthalmic diseases exhibit substantial heterogeneity in experimental models, light stimulation parameters (e.g., wavelength, irradiance, and energy density), and outcome measures. This variability limits the generalizability and reproducibility of the findings and hinders the establishment of standardized guidelines for PBM application in ocular conditions. Systematic reviews and meta-analyses on the effectiveness of PBM in ophthalmic diseases have reported some positive effects, particularly in clinical settings focusing on AMD (25, 26). Disease-specific literature reviews have been conducted separately (14, 21, 24); however, a comprehensive meta-analysis that systematically integrates these findings is required. A direct comparison of treatment outcomes across studies is difficult considering the wide variability in disease models, intervention protocols, and endpoints. To address this issue, it may be helpful to classify outcome measures into consistent and biologically meaningful domains that align with the primary therapeutic targets of PBM in ophthalmology. This classification into the morphological, visual function, and inflammatory domains may facilitate the integration of heterogeneous data in a more organized and interpretable manner. These categories correspond to the primary mechanisms through which PBM exerts its therapeutic effects and are closely aligned with the fundamental pathophysiological processes in ophthalmic diseases, including tissue degeneration, functional vision loss, and chronic inflammation. This framework increases comparability between studies and provides a basis for a more sophisticated interpretation of the therapeutic effects of PBM under various experimental conditions. Addressing the limitations of the existing fragmented literature can serve as the foundation for a more integrated and conceptually coherent understanding of the therapeutic impact of PBM across various ophthalmic disease models.

Therefore, this systematic review aims to provide an integrated synthesis of the preclinical therapeutic effects of PBM in ophthalmology, categorized into morphological, visual function, and inflammatory outcome domains, thereby offering consolidated conclusions regarding its therapeutic potential.

### 1.1 Research questions

1. What is the efficacy of PBM in improving morphological, functional, and inflammatory outcomes in preclinical animal models of AMD, DR, glaucoma, and corneal injury?
2. How do variations in PBM parameters (e.g., wavelength, irradiance, energy density, and treatment duration) influence the therapeutic efficacy of PBM in animal models of ophthalmic diseases?

### 1.2 Objectives

This systematic review and meta-analysis aims to comprehensively evaluate the therapeutic effects of PBM in animal models of ophthalmic disease, focusing on three predefined outcome domains: (1) morphological outcomes, such as retinal thickness, photoreceptor integrity, and retinal ganglion cell survival; (2) visual functional outcomes, including optokinetic responses and electroretinographic measurements; and (3) inflammatory outcomes, assessed via cytokine expression and microglial activation. By systematically synthesizing existing preclinical evidence, this study will aim to identify consistent patterns of efficacy, clarify optimal PBM parameters, and assess the robustness of the outcome measures used in prior studies. The findings of this review will provide a consolidated scientific foundation for the design of future preclinical and clinical trials, support the translation of PBM into clinical ophthalmic practice, and advance the development of evidence-based guidelines for its therapeutic application in ophthalmology.

## 2 Methods

### 2.1 Protocol and registration

This systematic review protocol will be conducted in accordance with the Preferred Reporting Items for Systematic Reviews and Meta-Analyses (PRISMA) guidelines (Additional File 1) and has been registered in the PROSPERO database (Registration number: CRD420251054002). To preserve the originality of this review, we will conduct a thorough search of relevant electronic databases (PROSPERO, Cochrane Library, and Google Scholar) for similar meta-analyses, systematic reviews, and protocol studies. No completed or ongoing systematic reviews or meta-analyses have addressed the efficacy and safety of photobiomodulation in animal models of ophthalmic diseases.

### 2.2 Eligibility criteria

#### 2.2.1 Types of studies

This review will include both randomized and non-randomized controlled preclinical studies that evaluate the therapeutic effects of PBM in ophthalmic disease models. Both blinded and unblinded designs will be considered. Only studies published in English will be included. No restrictions will be imposed regarding date or publication status.

#### 2.2.2 Types of animal models

Preclinical *in vivo* investigations conducted using animal models of AMD, DR, glaucoma, and corneal injury will be included in this study. Relevant models will involve disease induction via genetic manipulation, pharmacological interventions, surgical procedures, or other experimental approaches. The selected animal models will include rats, mice, or rabbits, regardless of strain, sex, or age. Human and *in-vitro* studies will be excluded. Representative examples of animal disease models include the following:

- AMD: models using light-induced retinal degeneration or sodium iodate injection, which replicate retinal pigment epithelium damage and photoreceptor loss.
- DR: streptozotocin- -induced diabetic models or db/db mice that mimic hyperglycemia-induced retinal microvascular pathology.
- Glaucoma: microbead-induced ocular hypertension or episcleral vein cauterization, representing progressive retinal ganglion cell (RGC) loss and optic nerve damage.
- Corneal injury: models based on alkali burns, mechanical epithelial debridement, or suture-induced neovascularization that emulate inflammation, scarring, and wound healing processes.

To ensure translational relevance, the included models must reproduce pathophysiological features consistent with those of human ophthalmic disorders.

#### 2.2.3 Types of intervention

The intervention of interest will be PBM therapy applied to animal models of ophthalmic diseases. Eligible studies must involve light irradiation at wavelengths within the red-to-near-infrared spectrum (e.g., 600–1,100 nm). Any light source (e.g., LED, laser), irradiation method (e.g., transpalpebral, transcorneal), energy density (J/cm^2^), power density (mW/cm^2^), treatment frequency, or duration will be accepted without restrictions. Single- and repeated-dose protocols will be included. Studies combining PBM with other therapeutic interventions will be excluded unless PBM-specific effects can be clearly isolated. The timing of PBM administration relative to disease induction (e.g., preventive or therapeutic) will be extracted but will not serve as an exclusion criterion. Additionally, subgroup analyses will be performed based on the timing of PBM administration (preventive vs. therapeutic) to explore potential differences in treatment outcomes.

#### 2.2.4 Types of comparators

A study will be included if it incorporates a PBM-unexposed animal control group within a study design that also features an intervention group receiving PBM, thus allowing for a direct comparison. The appropriate control groups may include:

- Sham light irradiation (e.g., an identical device applied without active light emission) to control for nonspecific handling or thermal effects.
- Untreated disease model group, in which the disease is induced but no therapeutic intervention is applied.

Control interventions must be relevant to the disease model under investigation. Studies that lack a clearly defined control group or an appropriate control intervention, fail to provide adequate data for evaluating the PBM intervention relative to the control, or employ a study design that does not permit a valid comparative analysis will be excluded.

#### 2.2.5 Types of outcome measures

This review will include animal studies that evaluate the therapeutic effects of PBM on ophthalmic diseases. To facilitate comprehensive evaluation, the included outcome measures will be categorized into common and disease-specific outcomes:

- Commone outcomes: These include retinal structure (e.g., retinal thickness, retinal ganglion cell viability), the degree of photoreceptor survival, retinal/visual function, metabolic assessment (specifically focusing on ATP levels), the status of neuroinflammation, and oxidative stress.
- Disease-specific outcomes: These include intraocular pressure and nerve integrity (for glaucoma), vascular patterns/abnormalities and retinal cell death (for DR), and corneal structure, neovascularization, and nerve integrity, and corneal cell viability (specifically focusing on corneal epithelial and stromal cells) (for corneal injury).

Studies will be excluded if they lack extractable numerical data, are unrelated to ophthalmic diseases, involve human subjects, do not isolate PBM effects, or are duplicate or secondary analyses of previously included studies.

### 2.3 Information sources

The following electronic databases will be systematically searched for studies published up to June 30, 2025: PubMed/MEDLINE, EMBASE, Web of Science, and Scopus. Only the studies published in English will be considered. No restrictions will be applied to the date of publication. Additionally, the reference lists of all included studies and relevant systematic reviews will be manually screened to identify eligible articles that were not captured by electronic search.

### 2.4 Search strategy

A comprehensive literature search will be conducted to identify relevant preclinical studies evaluating the effects of PBM in animal models of ophthalmic diseases. The search will be performed in accordance with the PICO framework using the following search terms:

- Population: Animal models of ophthalmic diseases, represented by the terms (“age-related macular degeneration” OR “AMD” OR “diabetic retinopathy” OR “DR” OR “glaucoma” OR “corneal injury” OR “retinal degeneration” OR “retinal damage”)
- Intervention: Photobiomodulation therapy, including (“photobiomodulation” OR “low-level light therapy” OR “low-level laser therapy” OR “PBM” OR “LLLT”)
- Comparator: Studies must include a PBM-unexposed control group, which may consist of a sham treatment involving the application of an identical device without active light emission. The selected control group should be appropriate for investigating the specific ophthalmic conditions.
- Outcomes: Studies assessing the structural, visual, functional, or inflammatory outcomes in the evaluation of PBM’s therapeutic efficacy of PBM for ophthalmic diseases will be included. These outcomes are key indicators for determining the extent of treatment benefits in preclinical models.
- Preclinical filter: (“animal model” OR “preclinical” OR “mice” OR “mouse” OR “rat” OR “rodent” OR “*in vivo*” OR “rabbit”)
  Manual searches of the reference lists of the included articles and relevant reviews will be conducted to identify studies that may have been missed during the electronic database search. No restrictions will be placed on the publication year or study location; however, only studies published in English will be included.

### 2.5 Study selection

Two authors will independently screen the titles, abstracts, and full texts of the identified studies based on the pre-defined inclusion and exclusion criteria. Any discrepancies will be resolved through discussion or adjudication by a third author. During the title and abstract screening phase, studies will be excluded in the following order of priority: (1) those not involving animal models of ophthalmic diseases; (2) those without PBM therapy intervention; and (3) review articles, conference abstracts, or commentaries. In the full-text screening phase, studies will be excluded if they (1) involve combination therapies, (2) lack access to the full text, (3) present outcome data in nonspecific or non-extractable formats, (4) are not peer-reviewed, (5) do not report the number of animals used, (6) are case studies, (7) lack a control group.

### 2.6 Data extraction

Two researchers will independently extract data from the included studies based on the pre-defined inclusion and exclusion criteria. Numerical data will be extracted from tables, the main text, or figure legends, wherever available. If data are presented only in graphical form, values will be digitized using the PlotDigitizer software (version 1.3) and digital rulers. Discrepancies between the two researchers will be resolved through discussion or consultation with a third researcher. In cases of missing or unclear data, we will contact the corresponding authors via email. If no response is received within 2 weeks, up to two follow-up emails will be sent.

### 2.7 Risk of bias assessment

The Risk of bias in the included studies will be assessed using the Systematic Review Centre for Laboratory Animal Experimentation (SYRCLE) risk-of-bias tool, which consists of 10 domains adapted from the Cochrane Risk of Bias (RoB) tool and is tailored for animal studies (27, 28). Two reviewers will independently evaluate each included study across the following domains: (1) sequence generation, (2) baseline characteristics, (3) allocation concealment, (4) random housing, (5) blinding of caregivers/investigators, (6) random outcome assessment, (7) blinding of outcome assessors, (8) incomplete outcome data, (9) selective outcome reporting, and (10) other sources of bias. Each domain will be rated as having a low, high, or unclear risk of bias, according to the criteria provided in the SYRCLE manual. Disagreements will be resolved through discussion or by consultation with a third reviewer.

### 2.8 Data analysis

All statistical analyses will be conducted using JASP (version 0.19.0.0) and STATA (version 17) depending on the functions required for each analysis. A meta-analysis will be performed when at least two studies report sufficiently similar outcomes; otherwise, a narrative synthesis will be performed. When sufficient data are available, subgroup analyses will be conducted to explore sources of heterogeneity. These will include comparisons based on the ophthalmic disease model (AMD, DR, glaucoma, or corneal injury), PBM intervention parameters including light wavelength (e.g., red vs. near-infrared wavelengths), energy density (low vs. high) PBM device type (LED vs. laser), and exposure duration (≤2 weeks vs. > 2 weeks), treatment frequency (e.g., number of applications per day or week), administration protocol (single- vs. repeated-dose), animal species (e.g., mouse, rat, rabbit), and the route of light delivery (transpalpebral, transcorneal, transscleral, or systemic).

Effect measures for continuous outcomes (e.g., retinal thickness, intraocular pressure, and ATP levels) will be calculated using either mean differences (MDs) or standardized mean differences (SMDs), each with 95% confidence intervals (CIs), depending on whether the measurements are reported in consistent units across the studies. Dichotomous outcomes (e.g., response rate and incidence of adverse effects) will be analyzed using odds ratios (ORs), risk ratios (RRs), risk differences (RDs), and 95% CIs. When appropriate, effect sizes will be converted into a common metric to facilitate pooling. Owing to the expected heterogeneity in disease models, species, and PBM protocols, a random-effects model will be used as the primary analytical method (29, 30). For comparative purposes, a fixed-effects model may be additionally applied in sensitivity analyses involving homogeneous subsets of studies such as the same disease model or species with low heterogeneity.

Heterogeneity will be assessed using Cochran’s Q test (*p* < 0.10 considered significant) and the I^2^ statistic, which will be interpreted as low (<40%), moderate (30–60%), or substantial (>50%). In studies with multiple PBM intervention groups, each group and its corresponding controls will be treated as separate comparisons. If a single control group is used for multiple interventions, it will be proportionally divided to avoid unit-of-analysis errors. Sensitivity analyses will be conducted using a leave-one-out approach, excluding individual studies with a high risk of bias, to examine their influence on the overall effect estimates. If ten or more studies are included in a given analysis, publication bias will be assessed through visual inspection of funnel plots and Egger’s regression test (*p* < 0.05 indicating potential bias). Where asymmetry suggests small study effects, a trim-and-fill analysis will be considered to evaluate the impact of missing studies on the pooled estimates. Furthermore, Kendall’s *τ* rank correlation and the Fail-safe N test will be employed to identify and measure the potential impact of unpublished studies on the overall results. If sufficient data are available, meta-regression analyses or meta-ANOVAs will be performed to investigate the continuous and categorical moderators, including treatment duration, total dose, and PBM energy density. A restricted maximum likelihood approach will be employed to estimate the model parameters. The overall strength and certainty of the evidence will be assessed using the Grading of Recommendations Assessment, Development and Evaluation (GRADE) approach. This will include evaluations of study limitations, consistency of results, directness of evidence, precision of estimates, and risk of publication bias.

## 3 Discussion

This study aims to systematically evaluate the therapeutic effects of PBM in preclinical animal models of four major ophthalmic conditions: AMD, DR, glaucoma, and corneal injury. Specifically, it seeks to assess both structural and functional outcomes and clarify how PBM efficacy varies according to disease type and stimulation parameters. These conditions will be included because they represent clinically important ophthalmic diseases and injury models associated with a substantial global burden of visual impairment and blindness and encompass diverse pathological contexts for evaluating the therapeutic effects of PBM. They are particularly prevalent in the elderly population and contribute to the continuously increasing global disease burden.

### 3.1 Summary of main findings

This systematic review and meta-analysis will synthesize preclinical evidence for the therapeutic effects of PBM in animal models of AMD, DR, glaucoma, and corneal injury. The main findings will provide a comprehensive assessment of the efficacy of PBM in improving structural and functional outcomes (e.g., visual function, tissue pathology, and inflammatory markers) across these disease models. Additionally, the review will identify patterns in treatment response based on disease type and stimulation parameters (e.g., wavelength, irradiance, and energy density). Therefore, this study is meaningful in elucidating the potential of PBM as a non-invasive therapeutic strategy and in supporting the development of standardized guidelines for clinical application in ophthalmology (14).

### 3.2 Current preclinical evidence on the therapeutic effects of PBM in ophthalmic diseases

PBM has shown promising therapeutic potential in AMD, DR, glaucoma, and corneal injury. Specifically, PBM attenuates structural degeneration in the outer retinal layers and reduces photoreceptor loss (31, 32). Moreover, preclinical investigations report that PBM contributes to the preservation of retinal integrity and visual function, particularly during the early and intermediate stages of disease progression (32, 33). In DR, several hallmark pathological features, including retinal hyperpermeability, capillary degeneration, and neuroretinal dysfunction, are substantially attenuated by PBM therapy (34, 35). Furthermore, long-term application of PBM has been found to markedly suppresses the progression of microvascular lesions and stabilizes visual performance, underscoring its potential role in slowing disease advancement (15). In the context of glaucoma, evidence from animal studies has highlighted the neuroprotective properties of PBM, particularly its ability to protect retinal ganglion cells and preserve optic nerve architecture. PBM has been consistently associated with the preservation of visual function under glaucomatous conditions (36). Corneal injury has similarly been shown to benefit from PBM therapy, which promotes epithelial wound healing, reduces stromal fibrosis and edema, and enhances corneal clarity (17, 37, 38). In addition, repeated applications have been shown to alleviate corneal discomfort and facilitate structural restoration of damaged tissue (39). This review will provide a comprehensive evaluation of the effectiveness and effect size of PBM in ophthalmic diseases by synthesizing results across disease types and animal models. This study will offer systematic insight into the preclinical evidence supporting the therapeutic benefits of PBM in various experimental models. Furthermore, by examining whether PBM produces consistent effects across diverse animal models, disease types, and irradiation parameters, this review will assess the consistency and reliability of its therapeutic efficacy. Collectively, these analyses contribute to a better understanding of PBM as a non-invasive intervention capable of attenuating key pathological features across a range of ophthalmic disease models.

### 3.3 Mechanistic interpretation of PBM effects in ophthalmic disease-related outcomes

This review will summarize the mechanisms of action of PBM by comparing different ophthalmic disease models and identifying the shared or common therapeutic pathways. To this end, outcome measures will be broadly categorized into three domains—morphological, visual functional, and inflammatory—based on the common pathophysiological features observed in AMD, DR, glaucoma, and corneal injury. As discussed in subsequent sections, this classification is intended to capture outcome measures that directly correspond to the molecular mechanisms through which PBM exerts its therapeutic effects in ophthalmic diseases.

#### 3.3.1 Morphological outcomes

PBM has been shown to preserve the retinal structural integrity in various ophthalmic disease models. This effect is primarily attributed to mitochondrial activation, which increases ATP production and thereby restores cellular energy metabolism (40, 41). Elevated energy availability supports the survival of photoreceptors and RGC. PBM also reduces oxidative stress by limiting reactive oxygen species accumulation and upregulating endogenous antioxidant enzymes, such as superoxide dismutase and catalase (42, 43). Moreover, although direct evidence from ophthalmic models is limited, several preclinical studies have demonstrated that PBM can activate cell survival pathways such as PI3K/Akt and ERK1/2, while concurrently suppressing pro-apoptotic signaling cascades including the GSK3β/Bax axis (44–46). These molecular effects have been associated with the increased expression of anti-apoptotic proteins (e.g., Bcl-2) and reduced expression of pro-apoptotic markers (e.g., Bax and caspase-9) (47). This evidence implies that PBM may promote neuroprotective signaling and inhibit RGC apoptosis in ophthalmic tissues, especially under degenerative conditions such as glaucoma.

#### 3.3.2 Visual functional outcomes

Evidence suggests that PBM improves visual function, as demonstrated by the increased amplitudes of electroretinography a- and b-waves, which reflect the preserved activity of photoreceptors and inner retinal neurons (32, 40, 48). Notably, similar neuroprotective effects have been observed in models of neurological disorders, where PBM enhanced the expression of BDNF or GDNF through activation of the ERK/CREB signaling pathway (49, 50). Through these molecular mechanisms, PBM may elicit neuroprotective responses in retinal neurons, including photoreceptors and retinal ganglion cells, by supporting synaptic stability, axonal regeneration, and neuronal survival.

#### 3.3.3 Inflammatory outcomes

Chronic inflammation is a central pathological process in several ophthalmic diseases. PBM exerts anti-inflammatory effects by downregulating proinflammatory cytokines such as TNF-*α*, IL-1β, and IL-6, as well as glial activation markers including Iba-1 and GFAP (18, 51, 52). These anti-inflammatory actions are mediated through the suppression of NF-κB and MAPK signaling pathways, while the activation of the Nrf2 pathway induces the expression of antioxidant genes such as HO-1 and NQO1 (53–55). By reducing inflammatory signaling and enhancing antioxidant defenses, PBM contributes to the maintenance of immune homeostasis and prevents secondary tissue damage caused by chronic inflammation. Furthermore, inflammatory responses involve the release of multiple cytokines, among which the upregulation of VEGF, a principal pro-angiogenic mediator, plays a pivotal role in driving pathological neovascularization in ophthalmic diseases. PBM reduces VEGF expression and vascular permeability, thereby stabilizing the blood-retinal barrier and minimizing retinal edema and tissue injury (56, 57). Based on these mechanisms, similar vascular protective effects have been observed in models of diabetic retinopathy and retinopathy of prematurity (15, 58). Collectively, these findings suggest that PBM may exert multifaceted regulatory effects on both the inflammatory and angiogenic pathways in ophthalmic diseases.

### 3.4 Clinical implication

Currently, PBM therapy is emerging as a non-invasive treatment in ophthalmology, especially for diseases such as dry AMD (20, 21). Several medical devices, such as Valeda and SightRetain, have been developed and approved for clinical use, showing a growing interest in and validation of PBM in ophthalmology. Therefore, the findings of this review are expected to highlight the potential of PBM as a non-invasive, neuroprotective treatment for AMD, glaucoma, DR, and corneal injury. By identifying consistent therapeutic effects and optimal PBM parameters in the preclinical models, this study may provide a basis for the application of PBM in clinical practice. This will support the development of evidence-based treatment protocols and guide future clinical trials aimed at expanding therapeutic options beyond current invasive or pharmacological interventions.

### 3.5 Limitations and future direction

This study has some limitations. First, there is considerable variability in animal species, disease models, disease types, and efficacy biomarkers among the included studies. Second, differences in PBM parameters (e.g., wavelength, irradiance, energy density, and frequency of application), as well as in outcome assessment methods, are likely to introduce substantial heterogeneity. This heterogeneity may limit the comparability and pooling of the results. Additionally, the included studies may have small sample sizes and may lack blinding, which could introduce bias. Future studies should focus on standardizing PBM protocols and exploring long-term functional outcomes. Comparative studies across different ophthalmic diseases are essential to further refine the therapeutic targets and optimize treatment strategies.

## Glossary

PBM: Photobiomodulation
LLLT: Low-Level Light Therapy
AMD: Age-related Macular Degeneration
DR: Diabetic Retinopathy
PRISMA-P: Preferred Reporting Items for Systematic Review and Meta-Analysis Protocols
SYRCLE: Systematic Review Centre for Laboratory Animal Experimentation
SD: Standard Deviation
SEM: Standard Error of the Mean
RoB: Risk of Bias
SMD: Standardized Mean Difference
VEGF: Vascular Endothelial Growth Factor
CFH: Complement Factor H
ATP: Adenosine Triphosphate
4-HNE: 4-Hydroxy-2-Nonenal
MDA: Malondialdehyde
RGC: Retinal Ganglion Cell
ROS: Reactive Oxygen Species
ERG: Electroretinogram
OKR: Optokinetic Response
BDNF: Brain-Derived Neurotrophic Factor
GDNF: Glial cell line-Derived Neurotrophic Factor
MD: Mean Difference
CI: Confidence Interval
OR: Odds Ratio
RR: Risk Ratio
RD: Risk Difference
